# “Pre-Existing and New-Onset Cognitive Impairment in Patients with COVID-19”

**DOI:** 10.1192/j.eurpsy.2022.104

**Published:** 2022-09-01

**Authors:** K. Krysta

**Affiliations:** Medical University of Silesia, Department Of Rehabilitation Psychiatry, Katowice, Poland

**Keywords:** Neuropsychiatric symptoms, cognitive functions, Covid-19

## Abstract

Recently, many reports have been available related to neuropsychiatric complications in the course of COVID-19 infection, and its long-term consequences. Many of them are related to psychological factors, such as: isolation, change of existing habits, fear of getting sick and dying, fear of losing a job and fears related to the financial situation, which resulted in an increase in the occurrence of diseases and mental disorders such as adaptation and anxiety disorders, depression. t has been also reported that treatment in conditions of intensive care unit lead to high prevalence of psychiatric disorders like PTSD in discharged patients. In our own studies we observed that patients with new appearing psychiatric symptoms were mostly those, who were professionally active, but did not work remotely. The time the patients spent at home had a negative impact on their well-being, they generally felt worse, their family life suffered more frequently. The most common cognitive symptoms, which are reported embrace: problems with short- and long-term memory, lack of mental clarity, feeling eclipsed, lightheaded, poor concentration, mental fatigue. We also present here a series of post-acute COVID cases from the examined population and provide a concise comparison of observed symptomatology with previous reports.

**Disclosure:**

No significant relationships.

